# Protocol for the identification of selected genes and haplotype analysis in soybean using next-generation sequencing

**DOI:** 10.1016/j.xpro.2025.104290

**Published:** 2025-12-23

**Authors:** Zhou Zhu, Zhixi Tian

**Affiliations:** 1 Yazhouwan National Laboratory, Sanya 572024, China; 2 University of Chinese Academy of Sciences, Beijing 100039, China

**Keywords:** bioinformatics, genetics, genomics, sequencing, plant sciences

## Abstract

Selected genes are genomic regions shaped by selection pressure and are often associated with important agronomic traits. Here, we present a protocol for identifying selected genes using genome resequencing data, followed by haplotype analysis of these genes. We describe steps for sequencing data collection and preprocessing, detection of genomic regions under selection, and haplotype construction based on sequence variation. The selected genes and haplotypes identified using this protocol provide insights into the genetic basis of soybean adaptation and improvement.

For complete details on the use and execution of this protocol, please refer to Zhu et al.^1^

## Highlights

• Protocol for genome-wide identification of selective sweeps in soybean
• Workflow for prioritizing selected genes and resolving key haplotypes
• Framework for cross-population mining of elite functional haplotypes

## Abstract (editor highlights)

Publisher’s note: Undertaking any experimental protocol requires adherence to local institutional guidelines for laboratory safety and ethics.

## Graphical abstract

**Figure:**
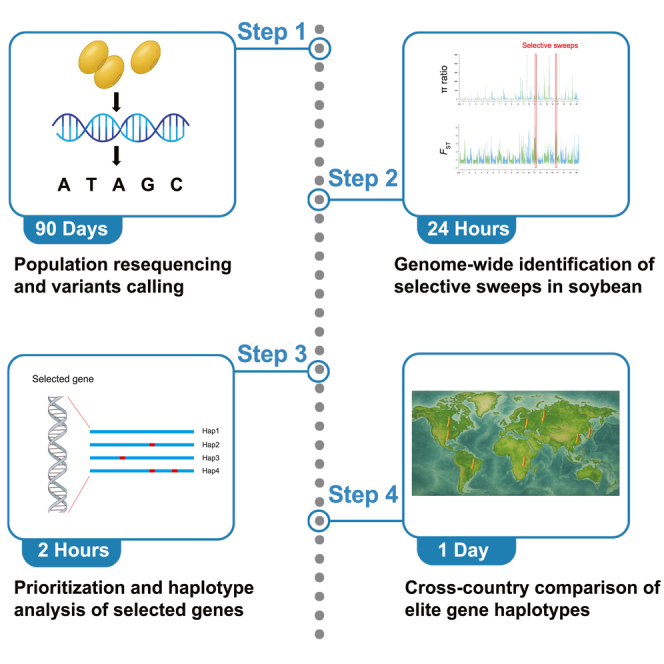

## Before you begin

This protocol leverages publicly available soybean resequencing data collected from multiple studies deposited in NCBI and the National Genomics Data Center of China. To ensure reliable detection of selected genes and accurate haplotype analysis, raw sequencing data must meet strict quality standards (e.g., high-quality genome resequencing data with sufficient sequencing depth, uniform coverage, and minimal missing sites to ensure completeness and accuracy). Detailed sample information, including accession identifiers, geographic origin, and population metadata, should be verified and curated prior to downstream analysis.

### Preparation 1: Data access and quality control

**Timing: Variable (affected by dataset size, internet speed, and computational resources)**

1. Obtain raw resequencing data:
  a. Collect published data or newly generated soybean whole-genome DNA sequencing data in FASTQ format from the sequencing facility.
  b. Confirm that metadata (sample identifiers, population origin, and experimental design) are provided together with the raw data.
  c. Ensure that sequencing was performed with sufficient coverage (average depth ≥ 5×) for downstream variant calling and haplotype analysis.

**CRITICAL:** Verify that the sequencing facility provides complete documentation on library preparation, sequencing platform, and read length, as inconsistencies can affect data comparability.

2. Verify data integrity:
  a. Check file completeness and integrity using checksum files (e.g., MD5).
  b. Ensure that all samples are accounted for and properly named according to the metadata sheet.
  c. Inspect a subset of FASTQ files with tools such as FastQC to confirm overall quality (base quality distribution, adapter contamination, and read length).

**CRITICAL:** Exclude or request replacement for samples that fail integrity or quality checks before proceeding to downstream processing.

### Preparation 2: Setting up the software environment

**Timing: 1 h**

3. Install all required software in a Linux environment, preferably using Conda. We applied multiple complementary population genetic metrics to ensure robust detection of selective signals:
  a. Weir and Cockerham’s *F*_ST_, calculated using VCFtools, to measure pairwise population differentiation.
  b. π ratio, computed with VCFtools, to identify regions exhibiting reduced nucleotide diversity indicative of selective sweeps.
  c. XP-CLR (Cross-Population Composite Likelihood Ratio) analysis, performed using the XP-CLR software, to detect cross-population selection based on allele frequency differentiation.
  d. RAiSD (Raised Accuracy in Sweep Detection), used to capture signatures of selective sweeps by modeling local distortions in the site frequency spectrum and linkage disequilibrium.

# environment.yml

name: python_env

channels:

 - bioconda

 - conda-forge

 - defaults

dependencies:

 - python=3.7

 - bwa=0.7.18

 - trimmomatic=0.39

 - samtools=1.20

 - gatk4=4.2

 - vcftools=0.1.16

 - xpclr

 - beagle=5.4

 # Utilities

 - wget

 - unzip

 # Note: RAiSD is NOT available through conda.

 # mkdir RAiSD && cd RAiSD

 # wget https://github.com/alachins/raisd/archive/master.zip

 # unzip master.zip

 # cd raisd-master

 # chmod +x install-RAiSD.sh

 # ./install-RAiSD.sh

**CRITICAL:** Ensure that the following software versions are compatible to prevent conflicts during analysis. When version mismatches occur or dependencies cannot be resolved, install individual tools in separate Conda environments.

### Innovation

This protocol introduces several advancements over existing approaches for identifying selected genes in soybean.

1. Integration of multiple algorithms.^2^^,^^3^^,^^4^^,^^5^ This workflow combines results from several established algorithms rather than relying on a single detection method, which reduces bias and improves the reliability of identifying genomic regions under selection.
2. Customized gene extraction. Tailored scripts are used to automatically extract genes located within candidate intervals, providing a direct and efficient link between statistical signals and biological targets.
3. Haplotype-level utilization analysis. By leveraging a high-density variation map and detailed sample metadata, the protocol characterizes haplotype structures for each selected gene and evaluates their distribution across populations from different geographic regions.

Together, these innovations establish a reproducible and versatile workflow that connects selection signals with functional haplotype diversity, offering insights into soybean domestication and breeding.^6^

## Key resources table

**Table:** 

REAGENT or RESOURCE	SOURCE	IDENTIFIER
**Deposited data**		

Soybean DNA sequencing data	Lu et al.^7^	NGDC BioProject: PRJCA001691
Soybean DNA sequencing data	Liu et al.^8^	NGDC BioProject: PRJCA002030
Soybean DNA sequencing data	Yang et al.^9^	NGDC BioProject: PRJCA002554
Soybean DNA sequencing data	Kajiya-Kanegae et al.^10^	BioProject: PRJDB7250
Soybean DNA sequencing data	Kajiya-Kanegae et al.^10^	BioProject: PRJDB7386
Soybean DNA sequencing data	Kajiya-Kanegae et al.^10^	BioProject: PRJDB7786
Soybean DNA sequencing data	N/A	BioProject: PRJEB1942
Soybean DNA sequencing data	N/A	BioProject: PRJEB31453
Soybean DNA sequencing data	N/A	BioProject: PRJNA175477
Soybean DNA sequencing data	N/A	BioProject: PRJNA227063
Soybean DNA sequencing data	N/A	BioProject: PRJNA243933
Soybean DNA sequencing data	N/A	BioProject: PRJNA248222
Soybean DNA sequencing data	Fang et al.^11^	BioProject: PRJNA257011
Soybean DNA sequencing data	N/A	BioProject: PRJNA274295
Soybean DNA sequencing data	Torkamaneh and Belzile^12^	BioProject: PRJNA287266
Soybean DNA sequencing data	Valliyodan et al.^13^	BioProject: PRJNA289660
Soybean DNA sequencing data	N/A	BioProject: PRJNA291452
Soybean DNA sequencing data	N/A	BioProject: PRJNA294227
Soybean DNA sequencing data	N/A	BioProject: PRJNA295763
Soybean DNA sequencing data	N/A	BioProject: PRJNA356132
Soybean DNA sequencing data	N/A	BioProject: PRJNA383915
Soybean DNA sequencing data	N/A	BioProject: PRJNA384190
Soybean DNA sequencing data	Fang et al.^11^	BioProject: PRJNA394629
Soybean DNA sequencing data	N/A	BioProject: PRJNA449253
Soybean DNA sequencing data	N/A	BioProject: PRJNA484078
Soybean DNA sequencing data	N/A	BioProject: PRJNA552939
Soybean DNA sequencing data	Kim et al.^14^	BioProject: PRJNA555366
Soybean DNA sequencing data	N/A	BioProject: PRJNA597660
Soybean DNA sequencing data	N/A	BioProject: PRJNA639876
Soybean DNA sequencing data	N/A	BioProject: PRJNA681974
Soybean DNA sequencing data	N/A	BioProject: PRJNA743225

**Software and algorithms**		

BWA 0.7.18-r1243-dirty	Li and Durbin^15^	https://sourceforge.net/projects/bio-bwa/
Trimmomatic 0.39	usadellab	https://github.com/usadellab/Trimmomatic
SAMtools 1.20	Li et al.^16^	https://sourceforge.net/projects/samtools/
GATK v.4.2	McKenna et al.^17^	https://github.com/broadinstitute/gatk/
VCFtools 0.1.16	Danecek et al.^18^	https://vcftools.github.io/man_latest.html
RAiSD v.2.9	Alachiotis and Pavlidis^5^	https://github.com/alachins/raisd
BEAGLE v.5.4	Pook et al.^19^	https://faculty.washington.edu/browning/beagle/
Xpclr	Vatsiou et al.^2^	https://github.com/hardingnj/xpclr

**Other**		

Soybean ZH13.v2 reference genome	This paper	https://ngdc.cncb.ac.cn/soyomics/static/data/download/genome/ZH13.v2.fasta.gz
The code and scripts employed for the analyses in this study	This paper	https://github.com/sibs-zz/ScricptsForSoybean
Online platform for querying haplotype analysis of selected genes	This paper	https://ngdc.cncb.ac.cn/soyomics/breedingtips

## Step-by-step method details

### Alignment and variant calling

**Timing: 72 h**

**Figure 1:**
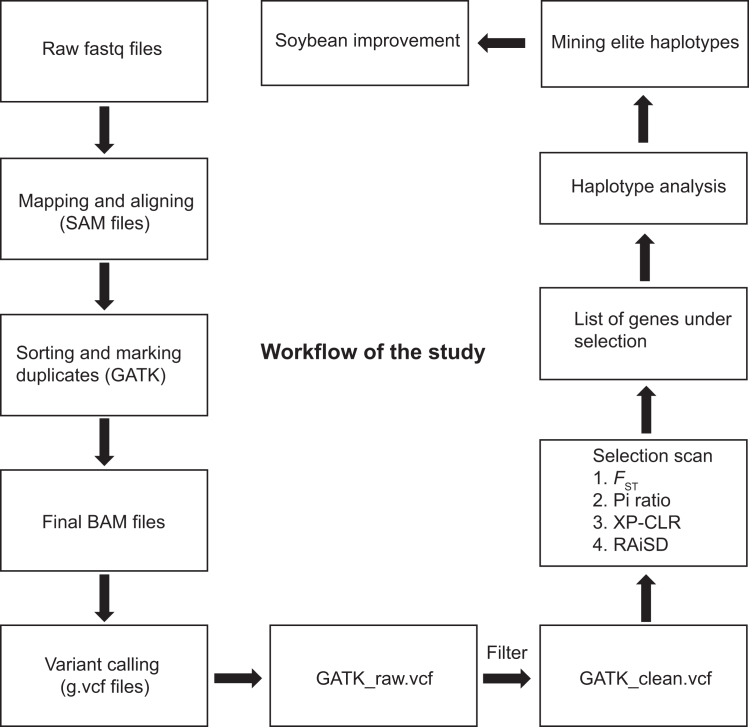
Overview of the data processing pipeline The workflow summarizes key processes including quality control, trimming, alignment to the reference genome, post-alignment processing, variant calling and downstream analysis.

This section focuses on processing raw sequencing reads, aligning them to the reference genome, and generating variant-ready BAM/VCF files for downstream selection and haplotype analysis. A workflow diagram (Figure 1) is also provided to illustrate the input/output relationships across all major steps.

1. Read preprocessing with Trimmomatic.
  a. Use Trimmomatic to remove adapter sequences and low-quality bases.
  b. Parameters: ILLUMINACLIP:TruSeq3-PE.fa:2:30:10 for adapter clipping, with additional trimming to ensure high-quality paired-end reads.
2. Read alignment with BWA-MEM.

Align cleaned paired-end reads to the soybean reference genome.

# Run BWA-MEM alignment

bwa mem \

 -t 4 \

 -M \

 -R "@RG\tID:sampleID\tPL:Illumina\tSM:sample" \

 reference.fa \

 sample_1_val_1.fq.gz \

 sample_2_val_2.fq.gz \

 > sample.sam

3. Convert SAM to BAM with Samtools.

# Convert SAM file to BAM format and filter out secondary alignments

samtools view \

 -F 0x100 \

 -Sb \

 sample.sam \

 > sample.bam

4. Sorting BAM with GATK SortSam.

# Sort BAM file by genomic coordinates using GATK SortSam

gatk SortSam \

 --INPUT sample.bam \

 --OUTPUT sample_sort.bam \

--SORT_ORDER coordinate

5. Duplicate marking with GATK MarkDuplicates.

# Mark duplicate reads using GATK MarkDuplicates

gatk MarkDuplicates \

 --INPUT sample_sort.bam \

 --OUTPUT sample_sort_MDup.bam \

 --METRICS_FILE sample_sort_MDup_metrics.txt \

 --VALIDATION_STRINGENCY SILENT \

 --OPTICAL_DUPLICATE_PIXEL_DISTANCE 100 \

 --ASSUME_SORT_ORDER coordinate \

 --CREATE_INDEX true

***Note:*** Parameter OPTICAL_DUPLICATE_PIXEL_DISTANCE should be set to 2500 for patterned flowcell-based sequencers.

6. Variant calling with GATK HaplotypeCaller.

# Call genomic variants and generate GVCF file using GATK HaplotypeCaller

gatk HaplotypeCaller \

 -R reference.fa \

 -I sample_sort_MDup.bam \

 -O sample.g.vcf \

 -ERC GVCF

### Joint genotyping and variant filtering

**Timing: Variable (weeks to months depending on sample size)**

7. Combine GVCFs.

Merge per-sample GVCFs into a single dataset.

# Combine individual GVCF files into a single cohort GVCF for joint genotyping

gatk CombineGVCFs \

 -R reference.fa \

 -V sample1.g.vcf \

 -V sample2.g.vcf \

 ... \

 -O cohort.g.vcf

8. Joint genotyping with GATK GenotypeGVCFs.

Perform cohort-level genotyping to produce a unified VCF file.

# Perform joint genotyping on the combined cohort GVCF to generate a final VCF file

gatk GenotypeGVCFs \

 -R reference.fa \

 -V cohort.g.vcf \

 -O cohort.vcf

9. Variant filtering.

Apply quality-based filtering to remove low-confidence variants.

# Apply quality-based filters to raw variants using GATK VariantFiltration

gatk VariantFiltration \

 -R reference.fa \

 -V cohort.vcf \

 --filter-expression "QD < 2.0 || FS > 60.0 || MQ < 40.0" \

 --filter-name "basic_snp_filter" \

 -O cohort_filtered.vcf

***Note:*** Joint genotyping and variant filtering are highly resource-intensive when dealing with thousands of high-depth resequencing samples. The process may take several weeks to months depending on sample size. It is strongly recommended to use a high-performance computing (HPC) environment with cluster scheduling, parallel execution, and GPU acceleration where available. For large datasets, splitting the genome into intervals and processing them in parallel can dramatically reduce runtime.

### *F*_ST_-based detection of selective sweeps

**Timing: 3 h**

This section identifies putative selective sweeps by contrasting populations with windowed *F*_ST_, standardizing scores, and mapping high-signal intervals to genes.

10. Inputs and population files.
  a. Prepare a filtered, high-quality cohort VCF (The VCF file used in this step is generated from Step 9. We applied additional quality control using VCFtools, filtering for biallelic single nucleotide polymorphisms (SNPs) with a minor allele frequency (MAF) ≥ 0.05 and missing rate ≤ 0.9).
  b. Create two text files listing sample IDs for each population (one ID per line), e.g., sample1.list and sample2.list. Ensure VCF sample names match the lists exactly.

# sample1.list (one sample ID per line)

SampleA

SampleB

SampleC

# sample2.list (one sample ID per line)

SampleD

SampleE

SampleF

11. Compute windowed *F*_ST_ with VCFtools.

# Calculate population differentiation (Fst) between two groups using VCFtools

vcftools \

 --vcf input.filtered.vcf \

 --weir-fst-pop sample1.list \

 --weir-fst-pop sample2.list \

 --out output_prefix \

 --fst-window-size 100000 \

 --fst-window-step 10000

# Example of output file: output_prefix.windowed.weir.fst

# CHROM BIN_START BIN_END N_VARIANTS WEIGHTED_FST MEAN_FST

# 1 1 100000 185 0.0245 0.0261

# 1 10001 110000 197 0.0283 0.0290

# 1 20001 120000 203 0.0327 0.0341

12. Clean and extract window statistics.

# Extract chromosome, window start, window end, and Fst value columns from the VCFtools output

tail -n +2 output_prefix.windowed.weir.fst \

 | cut -f1,2,3,5 \

 > output_prefix.fst.window

# Example of output file: output_prefix.fst.window

# 1 1 100000 0.0245

# 1 10001 110000 0.0283

# 1 20001 120000 0.0327

13. Z-score standardization across windows.

# Compute z-scores for windowed Fst values (add a 5th column 'z_score')

python zscore.py \

 output_prefix.fst.window \

 output_prefix.fst.window.zscore

# zscore.py

import sys

import pandas as pd

from scipy.stats import zscore

def calculate_zscore(input_file, output_file):

 # Load the data from the file

 data = pd.read_csv(input_file, sep=" ", header=None)

 # Ensure the data has at least four columns

 if data.shape[1] < 4:

  raise ValueError("Input file must contain at least four columns.")

 # Calculate z-score for the fourth column

 data['z_score'] = zscore(data.iloc[:, 3])

 # Save the data with the z-score to the output file

 data.to_csv(output_file, sep=' ', index=False, header=False)

if __name__ == "__main__":

 if len(sys.argv) != 3:

  print("Usage: python script.py <input_file> <output_file>")

 else:

  input_file = sys.argv[1]

  output_file = sys.argv[2]

  calculate_zscore(input_file, output_file)

14. Select outlier windows (e.g., Z ≥ 1.96).

# Extract candidate regions with significant selection signals (Z ≥ 1.96)

awk '$5 >= 1.96' output_prefix.fst.window.zscore | cut -d' ' -f1-3 \

 > output_prefix.fst.window.zscore.pos

# Example of output file: output_prefix.fst.window.zscore.pos

# 1 60001 160000

# 1 70001 170000

# 1 80001 180000

15. Normalize chromosome labels to match the GFF.

# Normalize chromosome names: add leading zero if <10 (e.g., Chr01, Chr02, ...)

awk '$1<10 {printf("Chr0%s %s %s\n",$1,$2,$3)} $1>=10 {printf("Chr%s %s %s\n",$1,$2,$3)}' \

 output_prefix.fst.window.zscore.pos \

 > output_prefix.fst.window.zscore.pos.norm

# Example of output file: output_prefix.fst.window.zscore.pos.norm

# Chr01 60001 160000

# Chr01 70001 170000

# Chr01 80001 180000

16. Map outlier intervals to genes (GFF overlap).

# Extract overlapping genes from GFF for the candidate windows

python GetGeneFromGFF.py reference.gff \

 output_prefix.fst.window.zscore.pos.norm \

 output_prefix.fst.window.zscore.pos.genes

# GetGeneFromGFF.py

import pandas as pd

import sys

def read_gff(gff_path):

 columns = ['seqname', 'source', 'feature', 'start', 'end', 'score', 'strand', 'frame', 'attributes']

 gff_df = pd.read_csv(gff_path, sep='\t', comment='#', names=columns)

 genes_df = gff_df[gff_df['feature'] == 'gene']

 return genes_df

def read_bed(bed_path):

 columns = ['chrom', 'start', 'end', 'name']

 bed_df = pd.read_csv(bed_path, sep=r'\s+', names=columns)

 return bed_df

def extract_genes_from_regions(gff_df, bed_df):

 extracted_genes = []

 for index, row in bed_df.iterrows():

 region_genes = gff_df[(gff_df['seqname'] == row['chrom']) & (gff_df['start'] <= row['end']) & (gff_df['end'] >= row['start'])]

 extracted_genes.append(region_genes)

 return pd.concat(extracted_genes) if extracted_genes else pd.DataFrame()

def main(gff_path, bed_path, output_path):

 gff_df = read_gff(gff_path)

 bed_df = read_bed(bed_path)

 genes = extract_genes_from_regions(gff_df, bed_df)

 genes.to_csv(output_path, index=False, sep='\t')

if __name__ == "__main__":

 gff_path, bed_path, output_path = sys.argv[1:4]

 main(gff_path, bed_path, output_path)

17. Extract unique gene IDs (candidate selected genes).

# Extract unique gene IDs from GFF annotation results (remove duplicates and clean attributes)

awk -F '\t' '{print $9}' output_prefix.fst.window.zscore.pos.genes \

 | grep -v attr \

 | sed 's/ID=//g' \

 | sed 's/;//g' \

 | sort -u \

 > output_prefix.fst.window.zscore.pos.genes.clean

# Example of raw gene list with duplicates (output_prefix.fst.window.zscore.pos.genes)

# SoyZH13_01G003210

# SoyZH13_02G045870

# SoyZH13_02G045870

# SoyZH13_03G100155

# SoyZH13_04G009900

# SoyZH13_05G201234

# SoyZH13_05G201234

# Example of cleaned, deduplicated output file (output_prefix.fst.window.zscore.pos.genes.clean)

# SoyZH13_01G003210

# SoyZH13_02G045870

# SoyZH13_03G100155

# SoyZH13_04G009900

# SoyZH13_05G201234

***Note:*** Ensure the same reference build is used for both the VCF and the GFF annotation file. Choice of window size and step (e.g., 100 kb/10 kb) will affect resolution; justify and report parameter choices.

### Nucleotide diversity (π) analysis and π-ratio-based sweep detection

**Timing: 3 h**

This section computes windowed nucleotide diversity (π) for two populations, derives the π ratio (popA/popB) on common windows, standardizes scores, and maps outlier intervals to genes.

18. Inputs and population files.
  a. Filtered cohort VCF: input.filtered.vcf (biallelic SNPs; recommended MAF/missingness filters).
  b. Two population lists with sample IDs: popA.list, popB.list (one ID per line).
  c. Gene annotation: reference.gff.
  d. Utility scripts: zscore.py for Z-score standardization; GetGeneFromGFF.py for interval to gene mapping.
19. Prepare per-population π jobs (windowed).

Create a shell script to run windowed π for each population list (100 kb windows; 100 kb steps shown as example).

# Generate per-population π calculation commands and save them into run_pi.sh

ls ∗.list | while read plist

do

 echo "nohup vcftools \

  --vcf input.filtered.vcf \

  --keep ${plist} \

  --window-pi 100000 \

  --window-pi-step 100000 \

  --out ${plist%.list} &"

 done > run_pi.sh && sh run_pi.sh

20. Extract window keys and intersect common windows.

# Extract common genomic windows shared between two populations (popA and popB)

grep -v BIN popA.windowed.pi \

 | awk -F ' ' '$1 && $2 && $3 {print $1"_"$2"_"$3}' \

 | sort \

 > A.keys

grep -v BIN popB.windowed.pi \

 | awk -F ' ' '$1 && $2 && $3 {print $1"_"$2"_"$3}' \ | sort \

 > B.keys

comm -12 A.keys B.keys > common.keys

21. Prefix π tables with window keys for joining.

# Tag each row with a composite window key

awk '{print $1"_"$2"_"$3, $0}' popA.windowed.pi > A.tag

awk '{print $1"_"$2"_"$3, $0}' popB.windowed.pi > B.tag

# Pull rows for common windows

while read k; do grep -w "$k" A.tag; done < common.keys > A.common

while read k; do grep -w "$k" B.tag; done < common.keys > B.common

22. Join tables, compute π ratio, and tidy.

***Note:*** In VCFtools windowed.pi, π is typically in column 4 (PI). Adjust field indices if your format differs.

# Compute π ratio per common window

paste A.common B.common \

 | awk '{print $2, $3, $4, $6, $12, $6/$12}' \

 > popA_vs_popB.piratio # CHR START END PI_A PI_B PIratio

# Sort by chromosome and start, then keep CHR START END PIratio

grep -v BIN popA_vs_popB.piratio \

 | sort -k1,1n -k2,2n \

 | awk '{print $1, $2, $3, $6}' \

 > popA_vs_popB.piratio.in

23. Z-score standardization and outlier selection.

# Calculate Z-scores for π ratio values

python zscore.py \

 popA_vs_popB.piratio.in \

 popA_vs_popB.piratio.in.zscore

# Extract genomic windows with significant Z-scores (Z ≥ 1.96)

awk '$5 >= 1.96 {print $1, $2, $3}' \

 popA_vs_popB.piratio.in.zscore \

 > popA_vs_popB.piratio.in.zscore.pos

24. Normalize chromosome labels to match the GFF (if needed).

# Normalize chromosome naming (add leading zeros, e.g., Chr01-Chr09)

awk '$1 < 10 {printf("Chr0%s %s %s\n", $1, $2, $3)} \

 $1 >= 10 {printf("Chr%s %s %s\n", $1, $2, $3)}' \

 popA_vs_popB.piratio.in.zscore.pos \

 > popA_vs_popB.piratio.in.zscore.pos.norm

25. Map outlier intervals to genes.

# Extract overlapping genes from GFF for candidate selective regions

python GetGeneFromGFF.py \

 reference.gff \

 popA_vs_popB.piratio.in.zscore.pos.norm \

 popA_vs_popB.piratio.in.zscore.pos.genes

# Extract and clean unique gene IDs from the GFF-derived table

awk '{print $9}' popA_vs_popB.piratio.in.zscore.pos.genes

 | grep -v attr \

 | sed 's/ID=//g' \

 | sed 's/;//g' \

 | sort -u \

 > popA_vs_popB.piratio.in.zscore.pos.genes.clean

***Note:*** Use identical window size and step for both populations; π ratios are meaningful only on common windows. Ensure consistent reference build and contig naming across VCF and GFF; normalize labels before overlap.

### RAiSD-based detection of selective sweeps

**Timing: 24 h**

This section runs RAiSD to compute the composite sweep statistic (*μ*), aggregates per-chromosome reports, standardizes scores, and maps high-signal intervals to genes.

26. Run RAiSD on the cohort.

# Detect genomic regions under selection using RAiSD

 ./raisd-master/bin/release/RAiSD \

 -n popA \

 -I input.filtered.vcf \

 -R \

 -A 0.95 \

 -S popA.list \

 -f

Output: per-chromosome reports named like RAiSD_Report.popA.1, RAiSD_Report.popA.2, …

27. Aggregate per-chromosome reports into one table.

# Merge RAiSD results from all chromosomes into a single summary file

: > popA.RAiSD.all

for f in RAiSD_Report.popA.∗

do

 chr=$(basename "$f" | awk -F. 'NF {print $NF}') awk '{printf "%.3f %.3f %.3f\n", $2, $3, $7}' "$f" \

| awk -v c="$chr" '{print c, $0}' \

 >> popA.RAiSD.all

done

28. Z-score standardization across genome-wide windows.

# Normalize RAiSD statistics using Z-score transformation

python zscore.py \

 popA.RAiSD.all \

 popA.RAiSD.all.zscore

29. Select outlier windows (e.g., Z ≥ 1.96).

# Extract genomic regions showing significant selection signals (Z ≥ 1.96)

awk '$5 >= 1.96 {print $1, $2, $3}' \

 popA.RAiSD.all.zscore \

 > popA.RAiSD.outliers.pos

Adjust the cutoff (e.g., top 1%/5%) according to study design.

30. Normalize chromosome labels to match the GFF (if needed).

# Normalize chromosome naming format for downstream gene mapping

awk '$1 < 10 {printf "Chr0%s %s %s\n", $1, $2, $3} \

 $1 >= 10 {printf "Chr%s %s %s\n", $1, $2, $3}' \

 popA.RAiSD.outliers.pos \

 > popA.RAiSD.outliers.norm

31. Map outlier intervals to genes and extract unique IDs.

# Extract candidate genes overlapping with significant selection regions

python GetGeneFromGFF.py \

 reference.gff \

 popA.RAiSD.outliers.norm \

 popA.RAiSD.outliers.genes

# Clean and deduplicate extracted gene list (SoyZH13 gene IDs)

awk '{print $9}' popA.RAiSD.outliers.genes \

 | grep -v attr \

 | sed 's/ID=//g' \

 | sed 's/;//g' \

 | sort -u \

 > popA.RAiSD.outliers.genes.clean

***Note:*** Use the same reference build and contig naming across VCF, RAiSD outputs, and reference.gff. For large cohorts/VCFs, parallelize by chromosome and run on HPC to mitigate I/O and CPU bottlenecks; keep intermediate files compressed and document software versions for reproducibility.

### XP-CLR-based detection of selective sweeps

**Timing: 24 h**

This section computes XP-CLR between two populations across all chromosomes, aggregates scores, standardizes them, and maps high-signal intervals to genes.

32. Inputs and population files.
  a. Filtered cohort VCF: input.filtered.vcf (biallelic SNPs; uniform MAF/missingness filters).
  b. Two population lists with sample IDs: popA.list, popB.list (one ID per line).
  c. Gene annotation: reference.gff.
  d. Utility scripts: zscore.py for standardization; GetGeneFromGFF.py for interval to gene mapping.
33. Run XP-CLR per chromosome.

Example parameters shown: LD cutoff 0.95, max 1000 SNPs per window, 100 kb window/step. Adjust to your design.

# Bash loop over chromosome IDs 1..20 (edit range for your genome)

for chr in $(seq 1 20); do

 xpclr \

  --out popA_vs_popB_chr${chr} \

  -Sa popA.list \

  -Sb popB.list \

  --input input.filtered.vcf \

  --chr ${chr} \

  --ld 0.95 \

  --maxsnps 1000 \

  --size 100000 \

  --step 100000

done

Output: one file per chromosome (e.g., popA_vs_popB_chr1, popA_vs_popB_chr2, …).

34. Concatenate chromosome outputs.

# Keep header from the first file, append non-header lines from the rest

head -1 popA_vs_popB_chr1 > popA_vs_popB_allchr

for chr in $(seq 1 20); do

 grep -v xpclr popA_vs_popB_chr${chr} >> popA_vs_popB_allchr

done

35. Extract genomic coordinates and XP-CLR score.

***Note:*** Typical XP-CLR outputs contain chromosome, window start/end, and the XP-CLR statistic; adjust column indices if your format differs.

# Extract chromosome position and XP-CLR score columns, remove headers and malformed lines

awk '{print $2, $3, $4, $12}' popA_vs_popB_allchr \

 | grep -v xpclr \

 | awk 'NF == 4' \

 > popA_vs_popB_allchr.score

36. Z-score standardization and outlier selection.

# Normalize XP-CLR scores using Z-score transformation

python zscore.py \

 popA_vs_popB_allchr.score \

 popA_vs_popB_allchr.score.z

# Identify genomic windows with significant XP-CLR signals (Z ≥ 1.96)

awk '$5 >= 1.96 {print $1, $2, $3}' \

 popA_vs_popB_allchr.score.z \

 > popA_vs_popB_allchr.outlier.pos

37. Normalize chromosome labels to match the GFF (if needed).

# Normalize chromosome naming format for consistency with reference genome (e.g., Chr01-Chr20)

awk '$1 < 10 {printf "Chr0%s %s %s\n", $1, $2, $3} \

 $1 >= 10 {printf "Chr%s %s %s\n", $1, $2, $3}' \

 popA_vs_popB_allchr.outlier.pos \

 > popA_vs_popB_allchr.outlier.pos.norm

38. Map outlier windows to genes and deduplicate IDs.

# Extract candidate genes overlapping with XP-CLR outlier regions

python GetGeneFromGFF.py \

 reference.gff \

 popA_vs_popB_allchr.outlier.pos.norm \

 popA_vs_popB_allchr.outlier.genes

# Clean and deduplicate extracted gene list (SoyZH13 gene IDs)

awk '{print $9}' popA_vs_popB_allchr.outlier.genes \

 | grep -v attr \

 | sed 's/ID=//g' \

 | sed 's/;//g' \

 | sort -u \

 > popA_vs_popB_allchr.outlier.genes.clean

***Note:*** Use the same reference build and consistent contig names across VCF, XP-CLR outputs, and reference.gff; normalize labels before overlap. Window size, step, LD cutoff, and --maxsnps strongly affect sensitivity and runtime; report chosen values and run sensitivity checks.

### Consensus integration of candidate genes across methods

**Timing: 5 min**

This section merges candidate gene lists from multiple detection approaches (e.g., *F*_ST_, π-ratio, XP-CLR, RAiSD). It supports flexible thresholds such as “identified by ≥1 method”, “≥2 methods”, or “≥3 methods”.

39. Inputs (one gene ID per line):

Make sure each file contains a single column of standardized gene IDs (no headers). If your IDs include attributes (e.g., ID=...;), clean them first as shown in earlier steps.

40. Streamlined code for merging gene lists.
  # Set threshold K (e.g., 1, 2, 3...)
  K=2
  # Merge all gene lists and compute support counts
  cat ∗.outlier.genes.clean \
   | sort \
   | uniq -c \
   > gene.counts.tmp
  # Genes supported by ≥K methods
  awk -v k=$K '{if ($1 >= k) print $2}' gene.counts.tmp \
   > consensus.k${K}.genes
  # Genes supported by exactly K methods (optional)
  awk -v k=$K '{if ($1 == k) print $2}' gene.counts.tmp \
   > consensus.eqk${K}.genes
  ***Note:*** This code concatenates all input lists, counts the number of methods supporting each gene, and outputs two files:
  a. consensus.kK.genes: genes detected by at least K methods.
  b. consensus.eqkK.genes: genes detected by exactly K methods.
    Ensure all lists use the same gene ID namespace (e.g., ZH13). If needed, map aliases to a canonical ID before merging. Record software versions and thresholds (window sizes, Z cutoffs). Store the exact shell commands in your workflow log or Makefile/Snakemake.

### Haplotype extraction and statistics

**Timing: 5 min**

This section extracts a target genomic interval from cohort VCFs, phases/genotypes (if needed), enumerates unique haplotypes per sample, and summarizes haplotype counts by geographic/population groups.

41. Inputs.
  a. Per-chromosome, bgzipped + tabix-indexed VCFs: .../vcf/soybean.∗.Chr∗.vcf.gz.
  b. Target interval: for example Chr2:50394718-50402312.
  c. Sample group lists (one sample ID per line), e.g., regionA.list, regionB.list, …
  d. Gene annotation (optional, for downstream mapping).
  e. haplo.py (the provided pysam script that prints per-sample haplotypes and labels them Haplotype1/2/...).

# haplo.py

import pysam

import sys

from collections import defaultdict

vcf = pysam.VariantFile(vcf_path)

haplotypes = defaultdict(lambda: [])

valid_positions = set()

for record in vcf:

 if all(record.samples[sample]['GT'] is not None for sample in record.samples):

  valid_positions.add(record.pos)

for record in vcf:

 if record.pos in valid_positions:

  for sample in record.samples:

   gt = record.samples[sample]['GT']

   haplotypes[sample].append(gt)

haplotypes_str = {sample: str(gt_list) for sample, gt_list in haplotypes.items()}

unique_haplotypes = defaultdict(lambda: [])

for sample, haplotype in haplotypes_str.items():

unique_haplotypes[haplotype].append(sample)

filtered_haplotypes = {k: v for k, v in unique_haplotypes.items() if len(v) >= 1}

sorted_haplotypes = sorted(filtered_haplotypes.items(), key=lambda x: len(x[1]), reverse=True)

for idx, (haplotype, samples) in enumerate(sorted_haplotypes, start=1):

 print(f"Haplotype{idx}: {haplotype}, Count: {len(samples)}")

 for sample in samples:

  print(f" Haplo{idx}: {sample}")

vcf.close()

42. Extract the interval across all per-chromosome VCFs.

# Pull the interval from every per-chromosome VCF and merge, then sort by POS

ls ../vcf/soybean.∗.Chr∗vcf.gz | while read v; do

 tabix "$v" Chr2:50394718-50402312

done | sort -k2,2n > interval.records

***Note:*** If you also need a VCF header, capture it once from any matching VCF.

zcat ../vcf/soybean.X.Chr2.vcf.gz | grep \# > header.vcf

43. Assemble a minimal interval VCF:

cat header.vcf interval.records > interval.raw.vcf

44. Phase/impute (recommended).

If your cohort isn’t fully phased or has sporadic missing genotypes, phase with Beagle on the interval.

beagle gt=interval.vcf out=interval_beagle

# Output: interval_beagle.vcf.gz

45. Call haplotypes per sample (across valid sites).
  python haplo.py interval_beagle.vcf.gz > interval.haplo
  The script.
  a. Loads all biallelic sites with complete genotypes across samples (“valid positions”).
  b. Builds a per-sample haplotype string over the valid sites.
  c. Prints unique haplotypes as Haplotype1, Haplotype2, … with member sample IDs.
    Example output (abridged).
    Haplotype1: [...] , Count: 4223
     Haplo1: SAMPLE_A01
     Haplo1: SAMPLE_A02
     ...
    Haplotype2: [...] , Count: 1786
     Haplo2: SAMPLE_B03
     ...
46. Count haplotypes by region/population.

For each region list (e.g., regionA.list), count how many listed samples fall into each haplotype.

# Count members of Haplotype1..Haplotype5 in regionA

for k in 1 2 3 4 5; do

 cat regionA.list \

 | while read sid; do grep -w "$sid" interval.haplo; done \

 | grep -w "Haplo${k}" \

 | wc -l

done

***Note:*** Phasing & missingness: Haplotype inference is sensitive to missing calls and phasing. Use Beagle (or comparable) and require consistent sample sets per locus. Ensure sample IDs and their case match exactly across VCF and region lists.

## Expected outcomes

This protocol yields a cohort-level, quality-controlled VCF suitable for selection scans, plus per-method genome-wide statistics and curated gene sets for downstream interpretation. From the four scan approaches (*F*_ST_, π-ratio, XP-CLR, RAiSD), researchers can expect chromosome-wide score tables (one row per window) that are Z-standardized within method, lists of outlier windows at predefined cutoffs, and interval-to-gene mappings that produce method-specific candidate gene lists. Consolidation across methods generates union and consensus gene sets (e.g., identified by ≥1, ≥2, or ≥3 methods).

**Figure 2:**
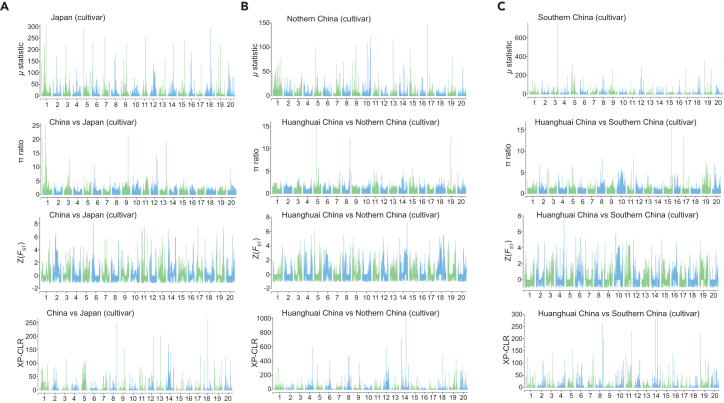
Genome-wide selection scans across soybean cultivars from different geographic regions (A) Comparisons between China cultivars and Japan cultivars. (B) Comparisons between Huanghuai China cultivars and Northern China cultivars. (C) Comparisons between Huanghuai China cultivars and Southern China cultivars. For each region, selection signals were identified using multiple statistics, including *μ* statistic, π ratio, standardized *F*_ST_, and XP-CLR. Each panel shows the distribution of selection signals along the 20 soybean chromosomes.

Figures should display genome-wide score tracks for all four methods. In a between-population contrast (e.g., China vs Japan, Figure 2A), expect discrete peaks where multiple methods co-localize, indicating putative selective sweeps; within-China regional contrasts typically reveal region-specific peaks (Figures 2B and 2C).

**Figure 3:**
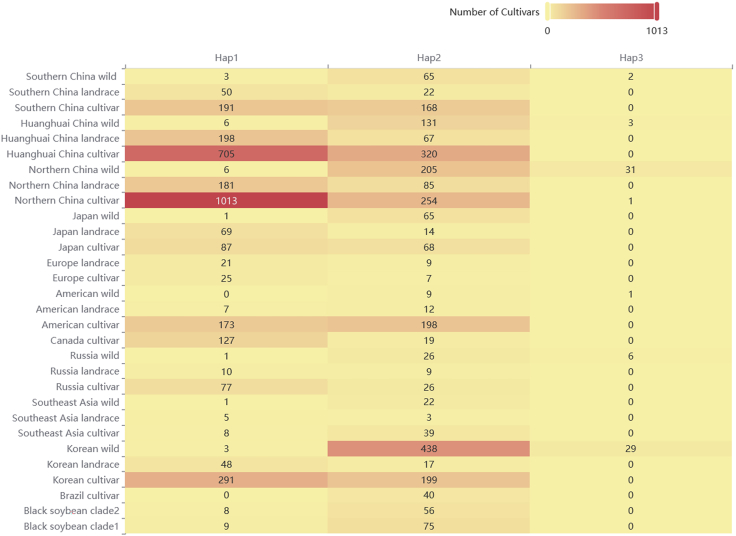
Distribution of *E2* (*SoyZH13_10G204600*) haplotypes among wild, landrace, and cultivated soybean groups across different geographical regions The heatmap illustrates the number of accessions carrying each *E2* haplotype (Hap1-Hap3) in diverse soybean populations, including wild, landrace, and cultivated groups from various countries and regions. The color gradient represents the number of cultivars, with darker red indicating higher frequency of the corresponding haplotype within a population.

Gene-based haplotypes were constructed from high-confidence SNPs using GATK for variant filtering and Beagle for genotype phasing and imputation. Haplotype analysis at candidate loci produces, for each interval, the number of unique haplotypes and a membership list of samples per haplotype. Aggregating by region (e.g., geographic groups within China) yields count tables or heatmaps that visualize haplotype utilization across populations (Figure 3) (more examples can be found in https://ngdc.cncb.ac.cn/soyomics/breedingtips). Typical outcomes include one or more predominant haplotypes per locus with minor alternatives; regionally enriched haplotypes are expected at loci flagged by multiple selection scans.

Deliverables include: per-method windowed statistics (with Z-scores), outlier window BED-like files, method-specific candidate gene lists, consensus gene sets at user-defined thresholds, and haplotype count matrices by region. Together, these outputs support narrative results (genome-wide peaks, convergent signals, and region-biased haplotypes) and figure panels illustrating whole-genome statistics for user-specified comparisons.

**Figure 4:**
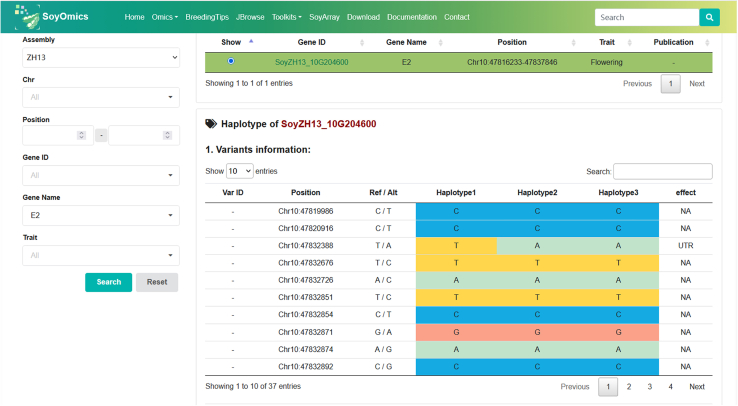
Screenshot of the online soybean haplotype query platform (SoyOmics) showing haplotype structure of the *E2* gene This figure illustrates the web interface developed for querying gene haplotypes in soybean populations. The example shown is for the *E2* gene, displaying three major haplotypes and their corresponding SNP variant positions across the gene region. Each column represents a haplotype, while colored cells indicate the allelic states (Ref/Alt) of the SNPs defining each haplotype.

Overall, this protocol enables the systematic identification of genomic regions and candidate genes under selection by integrating multiple population genetic statistics. By combining these complementary approaches, researchers can pinpoint loci that have undergone selection during domestication or adaptation to diverse environments. The subsequent haplotype analysis allows the characterization of advantageous haplotypes associated with desirable agronomic traits such as yield, flowering time, and stress tolerance. These insights can facilitate the discovery of elite alleles and haplotypes (Figure 4) that contribute to environmental adaptation and yield improvement, providing a foundation for molecular breeding and genetic improvement of soybean.

## Limitations

This protocol relies on heterogeneous resequencing datasets aggregated from multiple sources; differences in library preparation, sequencing platform, read length, and DNA quality can introduce batch effects that are not fully removed by standard QC and normalization. Uneven or sub-threshold coverage (<∼5×), high missingness, or inaccurate metadata (e.g., mislabeled geographic origin) reduce power and may produce spurious between-population contrasts. Resequencing data newly generated from the same batch of materials through the standardized pipeline of the same sequencing company ensure improved reliability and performance of this analytical workflow.

Reference-related issues—including misassemblies, repetitive/pericentromeric regions with low mappability, and annotation/version mismatches—can distort window statistics and gene mapping, particularly when contig names or builds are inconsistent across VCF and GFF files. Additionally, differences in reference genome assembly quality and annotation versions can result in inconsistent variant detection and may affect the interpretation of selective sweep signals and haplotype structure.

Selection-scan statistics have method-specific sensitivities and assumptions; demographic history (bottlenecks, structure, admixture/introgression) can mimic sweep-like signals, while strong LD or low recombination can inflate scores independent of recent selection. Window-based analyses are sensitive to parameter choices (window size, step, LD cutoff) and Z-score thresholding. Cross-method consensus increases robustness but may miss true positives detectable by only one approach, and it does not by itself resolve demographic confounding without explicit modeling or permutation-based calibration.

Haplotype inference depends on accurate genotypes and phasing. When phasing is imperfect or missingness is nonrandom, inferred haplotypes and their regional counts can be biased. The illustrative haplotype script retains only sites genotyped in all samples; in large cohorts this may discard informative variants and skew haplotype definitions toward common sites. Structural variants, copy-number variation, and presence/absence polymorphisms are largely invisible to SNP-based scans and may underlie genuine adaptation that this workflow will not detect.

## Troubleshooting

### Problem 1

Adapter contamination or poor base quality leads to low mapping rates. (step 1).

### Potential solution

Inspect reads with FastQC; increase trimming stringency (e.g., add SLIDINGWINDOW:4:20, MINLEN=36) and verify ILLUMINACLIP adapters match the library kit.

Users may observe messages such as.

• “Adapter sequence detected” or excessive adapter content in FastQC reports.
• “Per base sequence quality: FAIL/WARN” in FastQC.
• Low alignment rate (<70%) reported by BWA/SAMtools (e.g., “mapped: 65%”).

For each of these problems, we now provide clear solutions, including.

• Re-running adapter and quality trimming (e.g., with Trimmomatic or fastp).
• Verifying improvement in per-base quality and adapter removal using FastQC before alignment.
• Checking for overrepresented sequences or library preparation issues.

### Problem 2

SAM to BAM conversion errors or truncated files. (step 3).

### Potential solution

• Check disk space and ulimit; prefer samtools sort pipeline over intermediate SAM.
• Validate with samtools quickcheck and regenerate if corruption is detected.

### Problem 3

Joint genotyping is prohibitively slow or fails. (step 7–8).

### Potential solution

• Use GenomicsDBImport + GenotypeGVCFs instead of CombineGVCFs for large cohorts.
• Scatter by chromosome/interval; run on HPC with job arrays; keep I/O local (node-local SSD).
• Confirm consistent sample order and identical reference dictionary across all inputs.

### Problem 4

Windowed *F*_ST_ contains NA or unexpected BIN header rows. (step 11).

### Potential solution

• Ensure both population lists match VCF sample IDs; remove empty/typo lines.
• Apply uniform missingness/MAF filters; drop monomorphic windows.
• Strip non-data lines before downstream parsing as shown in the workflow.
• We have added a validation script that automatically checks input and output file formats for consistency and completeness.

#!/usr/bin/env python3

import sys

if len(sys.argv) != 2:

print("Usage: python clean_fst.py <windowed_fst.txt>", file=sys.stderr)

sys.exit(1)

infile = sys.argv[1]

with open(infile) as f:

 
next(f) # skip header line

 
for line in f:

 
if "NA" in line or "NaN" in line or "nan" in line:

 
continue # skip NA lines

 
fields = line.strip().split()

 
if len(fields) < 6:

 
continue

 
chrom = fields[0]

 
start = fields[1]

 
end = fields[2]

 
fst = fields[4] # WEIGHTED_FST

 
print(chrom, start, end, fst, sep="\t")

### Problem 5

π-ratio windows do not line up between populations. (step 19).

### Potential solution

• Use identical --window-pi and --window-pi-step for both groups.
• Intersect common windows (as scripted) before ratio calculation; avoid mixing different filters between groups.

### Problem 6

RAiSD outputs missing for some chromosomes or *μ* distribution looks abnormal. (step 26).

### Potential solution

• Verify chromosome naming matches VCF; run per-chromosome and aggregate with the provided loop.
• Confirm input MAF filters and SNP density.

### Problem 7

XP-CLR reports few or no windows/errors about insufficient SNPs. (step 33).

### Potential solution

• Reduce --maxsnps or increase window size; relax --ld threshold slightly if appropriate.
• Confirm population lists are non-overlapping and reasonably sized; ensure VCF is biallelic and filtered.

### Problem 8

Gene mapping yields zero hits. (All mapping steps using GetGeneFromGFF.py).

### Potential solution

• Normalize contig labels (e.g., Chr02 vs 2) before overlap; ensure GFF and VCF share the same build.
• Expand windows slightly to accommodate annotation boundaries; verify GFF column conventions.

### Problem 9

Haplotype script returns very few sites or no haplotypes. (step 47).

### Potential solution

• The script currently requires all samples to be non-missing at each site; relax this by allowing ≥95% call rate, or phase/impute with Beagle first.
• Split multiallelics with bcftools norm -m-any and maintain consistent sample sets across loci.

## Resource availability

### Lead contact

Requests for resources, data, and further information should be addressed to the lead contact, Zhou Zhu (zhuzhou@yzwlab.cn).

### Technical contact

Technical questions on executing this protocol will be answered by the technical contact, Zhou Zhu (zhuzhou@yzwlab.cn).

### Materials availability

This study did not generate new reagents.

### Data and code availability

The code generated during this study is publicly available on GitHub (https://github.com/sibs-zz/ScricptsForSoybean) and has been archived on Zenodo (DOI: https://doi.org/10.5281/zenodo.17784450).

## Acknowledgments

This work was supported by the Yazhouwan National Laboratory Project (2310ZX01), the National Natural Science Foundation of China (grant 32388201), the Xplorer Prize (to Z.T.), and the Taishan Scholars Program (to Z.T.). We thank Professor Guodong Wang (Institute of Genetics and Developmental Biology, Chinese Academy of Sciences) and Professor Dawei Xin (Northeast Agricultural University, Harbin, China) for assistance in this study.

## Author contributions

Z.T. supervised the project. Protocol and analysis pipeline were organized and contributed by Z.Z.

## Declaration of interests

The authors declare no competing interests.
